# Protocol to infer off-target effects of drugs on cellular signaling using interactome-based deep learning

**DOI:** 10.1016/j.xpro.2024.103573

**Published:** 2025-01-16

**Authors:** Nikolaos Meimetis, Douglas A. Lauffenburger, Avlant Nilsson

**Affiliations:** 1 Department of Biological Engineering, Massachusetts Institute of Technology, Cambridge, MA 02139, USA; 2 Department of Cell and Molecular Biology, SciLifeLab, Karolinska Institutet, 171 77 Stockholm, Sweden

**Keywords:** bioinformatics, health sciences, gene expression, systems biology

## Abstract

Drugs that target specific proteins often have off-target effects. We present a protocol using artificial neural networks to model cellular transcriptional responses to drugs, aiming to understand their mechanisms of action. We detail steps for predicting transcriptional activities, inferring drug-target interactions, and explaining the off-target mechanism of action. As a case study, we analyze the off-target effects of lestaurtinib on FOXM1 in the A375 cell line.

For complete details on the use and execution of this protocol, please refer to Meimetis et al.^1^

## Highlights

• This protocol preprocesses gene expression data for deep learning modeling
• Trains a model to predict drug-induced transcription factor activity on cell lines
• Infers drug-target interactions that explain the transcriptomics data
• Extracts and visualizes the mechanism of action of drugs’ off-target effects

## Abstract (editor highlights)

Publisher’s note: Undertaking any experimental protocol requires adherence to local institutional guidelines for laboratory safety and ethics.

## Graphical abstract

**Figure:**
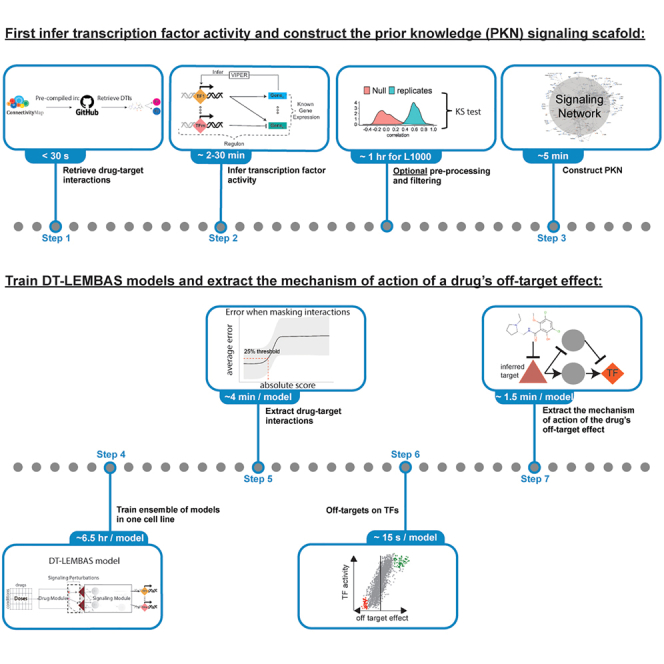

## Before you begin

This section includes the software installation as well as the collection of pre-compiled data used in this study to train and utilize a DT-LEMBAS model.^1^

DT-LEMBAS is an interactome-based artificial neural network model that takes as input log-transformed drug concentrations and predicts the induced activity of multiple transcription factors. DT-LEMBAS consists of two components: a drug layer that transforms input drugs to signaling perturbations on available target nodes (for a pre-defined drug-target space), and a signaling module (termed LEMBAS^2^), defined as a recurrent neural network and constraint to an existing prior knowledge signaling network (PKN), which takes the signaling input and propagates it in the intracellular network to predict transcription factor activity. Empirically, in our case studies and data^1^^,^^2^ we observed that at least ∼120 conditions are needed when training a predictive model using ∼100 TFs, and a Prior Knowledge Network (PKN) of ∼12 thousand interactions.

### Hardware requirement

Simple simulations using one model were performed on a Dell XPS 17 laptop with an Intel i9-11900h @4.9 GHz with 8 cores (16 logic processors) and 32 GB RAM. For convenience, ensemble training of multiple models and cross-validation was carried out on a single-threaded computer cluster (Intel Xeon CPU @ 2.60 GHz) that allowed job scheduling (using Slurm) with 16 parallel jobs. For extracting inferred drug-target interactions from the model for the specific linear drug module used in this study only the CPU version of PyTorch^3^ is required, and this results in an execution time of ∼3 min per model. For the purpose of generalization to custom-built drug-interaction modules, we have also implemented a version based on integrated gradients using the Captum library.^4^ This analysis uses the GPU version of PyTorch, requiring an execution time of ∼3.8 min per model for our case study. This method yields equivalent results as the CPU version but can be applied to custom-developed, non-linear drug modules (e.g., instead of using weight matrices one could use conventional feedforward neural networks with non-linear activation functions), with some minor tuning. Specifically, the step evaluating drug-target interactions can take any PyTorch model and generate a score for each output (e.g., target protein) with respect to the input (e.g., drug).

### Software installation

**Timing: ∼30 min**

For this case study, as well as the associated publication and its results, all models were expressed in and trained using the PyTorch framework^3^ (versions 1.10.2 & 1.12) in Python (version 3.6.13 & 3.8.8). Pre-processing and statistical analysis of the results were done in the R programming language (version 4.1.2). Visualization of results was done mainly using ggplot2^5^ and Cytoscape.^6^ For more information about the library versions used in the original study, visit the corresponding GitHub repository: https://github.com/Lauffenburger-Lab/DrugsANNSignaling.

The installation commands have been verified to work in Unix-based, macOS (with the exception of the GPU functionalities), and Windows operating systems. More information is available in the steps below.

1. Clone the code repository from https://github.com/Lauffenburger-Lab/DrugsANNSignaling

> git clonehttps://github.com/Lauffenburger-Lab/DrugsANNSignaling.git.

2. Install Anaconda on your personal computer (for cluster installation follow your cluster’s admin instructions).
  a. Download Anaconda from https://repo.anaconda.com/archive.
  b. Install the Anaconda Navigator following the default settings.
  c. Launch the Anaconda Navigator and open the terminal **or** directly open the Anaconda Powershell Prompt **or** (for Unix-based systems) open your command line.
  d. Direct with “cd” commands to the folder you want to run your case study (the cloned GitHub folder)
3. Create a conda environment and activate it to install Python DT-LEMBAS dependencies.

> conda create -n DTLembas

> conda activate DTLembas

4. Install Python libraries.

> conda activate DTLembas

> conda install -c conda-forge rdkit # It will also install numpy, pandas, and matplotlib

> conda install -c conda-forge scikit-learn #it will also install scipy

> pip install networkx

***Note:*** Installing rdkit will also install numpy, pandas, and matplotlib, while scikit-learn will also install scipy.

5. Install PyTorch framework (select the GPU option if GPU and CPU are both available in your operating system. macOS is not compatible with the GPU version).
  > conda activate DTLembas
  a. GPU+CPU version for CUDA 11.8 (for more information see https://pytorch.org/get-started/locally/).
    > conda install pytorch torchvision torchaudio pytorch-cuda=11.8 -c pytorch -c nvidia
  b. CPU-only version (for more information see https://pytorch.org/get-started/locally/).
    > conda install pytorch torchvision torchaudio -c pytorch
6. Install Captum for feature importance calculation:

> conda install captum -c pytorch

7. Install R and R-studio.
  a. Download R from https://cloud.r-project.org and install it.
  b. Download R-studio from https://posit.co/download/rstudio-desktop and install it.
8. Install necessary R packages (Inside R or RStudio terminal run the following commands).

>if(!require(("BiocManager", quietly = TRUE)){ > install.packages("BiocManager") # If not already installed >} > BiocManager::install(c("cmapR","rhdf5","dorothea","org.Hs.eg.db","hgu133a.db"))

>if(!require(("tidyverse", quietly = TRUE)){ > install.packages("tidyverse") # If not already installed >}

>if(!require(("ggplot2", quietly = TRUE)){ > install.packages("ggplot2") # If not already installed >}

> install.packages("ggrepel")

> install.packages("ggpubr")

> install.packages("doRNG")

> install.packages("doFuture")

***Note:*** For using R in computer clusters, cluster-specific commands may be required. However, there is a convenient alternative of creating an R conda environment using anaconda or miniconda. **A lot of the dependencies, such as tidyverse and ggplot2, are automatically installed this way.** Below we show how to create the R environment and install the remaining dependencies:

> conda create -n DTLembas_r_env

> conda activate DTLembas_r_env

> conda install -c r r-essentials

> conda install r-BiocManager

> conda install conda-forge::r-ggrepel

> conda install r-ggpubr

> conda install r-doRNG

> conda install r-doFuture

> R() # to open R to install Bioconductor packages and GitHub repos

> BiocManager::install(c("cmapR","rhdf5","dorothea","org.Hs.eg.db","hgu133a.db"))

9. Install the latest version of Cytoscape to visualize the inferred mechanism of action networks here: https://cytoscape.org.
10. Optionally, download the yFiles Layout Algorithms for Cytoscape here: https://www.yworks.com/products/yfiles-layout-algorithms-for-cytoscape.
11. Open the Cytoscape app and select `Apps>App>Install Apps From File Store` in the main menu bar.
12. Select the file you downloaded for the yFiles Layout Algorithms.

***Note:*** These layout options are useful for visualization and especially for our case studies for selecting the yFiles Organic Layout and yFiles Remove Overlaps (which removes overlaps between edges).

### File types and formats

The whole tutorial utilized three file types: i) CSV, ii) TSV, and iii) RDS. The RDS file format is a unique data type where R objects and variables of any kind can be saved into. For converting from and to RDS format you may use the RDS_to_CSV_TSV.R and CSV_TSV_to_RDS.R, respectively, in the preprocessing folder. It is also possible to load and save RDS files in Python (look at the **CSV_TSV_to_RDS.py** example in the preprocessing folder) by using the `pyreadr` package (https://ofajardo.github.io/pyreadr/_build/html/index.html).

The identifiers used for individual drugs follow the Simplified Molecular Input Line Entry System (SMILES) format. SMILES is a sequence of letters describing the 2D structure of a drug. Wherever a commercial drug name is used, this is only included for interpretability, as a unique chemical structure (SMILES) can have multiple commercial names.

The genes in this tutorial are annotated using gene names of the NCBI standard. Before you begin it is important to annotate your transcriptomic data with these gene names, as these are also used when inferring transcription factors’ activities.

For training the model, three main input files are required. Firstly, a PKN saved in TSV format with columns: source (i.e., the source node in a protein-protein interaction), target (i.e., the target node in a protein-protein interaction), direction, stimulation, inhibition, sources and references. Secondly, the input log-transformed drug concentrations are the main input of the whole model (the drug layer and the signaling module together), saved in a TSV file in matrix format (samples(N)xdrugs(D)), where the rows are named with any condition identifier and columns are named with the SMILES of the drugs. Finally, the predicted output of the model is saved in a TSV file in matrix format (NxTFs(T)), where rows have the same names as the input file, and the columns have the names of the TFs whose activity is going to be predicted.

### Data collection

**Timing: ∼30 s**

All the data required for this tutorial are automatically downloaded during the software installation when cloning the GitHub repository with the following command.

> git clonehttps://github.com/Lauffenburger-Lab/DrugsANNSignaling.git.

For users with their own data, this tutorial is designed to be flexible. You may substitute your own transcriptomic and drug-target interaction data for the analysis, provided it follows the same format as the file used in this tutorial (see Step 1). This allows for customized case studies and specific data exploration relevant to your research. Note that for your transcriptomic data, you need to annotate them with the corresponding gene symbols, in order to match the gene symbols (NCBI standard) of the prior knowledge regulatory network that is used.

For completing the tutorial using our preprocessed data, is provided in the preprocessing/preprocessed_data folder. It contains drug-target interaction, in the ***L1000_lvl3_A375-drugs_targets.tsv*** file, located in the TrainingValidationData folder; the input conditions for the model, i.e., the doses of the drugs tested in the experiment, in the ***L1000_lvl3_A375-conditions_drugs.tsv*** file, located in the TrainingValidationData folder; and the transcription factor activities of input conditions used to train the model, in the ***TrimmedFinal_l1000_allgenes_lvl3_tfs.tsv*** file, located in the TF_activities folder.

## Key resources table

**Table:** 

REAGENT or RESOURCE	SOURCE	IDENTIFIER
**Deposited data**		

L1000 Connectivity Map perturbational profiles from Broad Institute LINCS Center for Transcriptomics LINCS Pilot Phase I	https://www.ncbi.nlm.nih.gov/geo/query/acc.cgi?acc=GSE92742	GSE92742
Broad Institute Repurposing Hub	https://repo-hub.broadinstitute.org/repurposing#download-data	Drug information: version 3/24/2020
Pre-processed and pre-compiled data	https://github.com/Lauffenburger-Lab/DrugsANNSignaling	https://github.com/Lauffenburger-Lab/DrugsANNSignaling
Pre-processed and pre-compiled data	https://doi.org/10.5281/zenodo.14057135	https://doi.org/10.5281/zenodo.14057135
Trained cell line-specific ensembles of 50 models	https://doi.org/10.5281/zenodo.14057298	https://doi.org/10.5281/zenodo.14057298

**Software and algorithms**		

R programming language v.4.1.2	R Core Team and the R Foundation for Statistical Computing	https://www.r-project.org
Python programming language v.3.8.8	Python Software Foundation	https://www.python.org
PyTorch framework (versions 1.10.2 & 1.12)	Linux Foundation umbrella	https://pytorch.org
Cytoscape v.3.10.3	Cytoscape Team	https://cytoscape.org
Machine learning and downstream analysis algorithms for this protocol	https://github.com/Lauffenburger-Lab/DrugsANNSignaling	https://github.com/Lauffenburger-Lab/DrugsANNSignaling

## Step-by-step method details

Here, we describe step-by-step methods for training interactome-based deep learning models to simulate cellular transcriptional responses to drugs. This aims to infer off-target effects and to understand their downstream signaling activities. This protocol is divided into 7 steps: 1) retrieving pre-compiled drug-target interactions (DTIs) or using your own with the same file formats, 2) inferring transcription factor (TF) activity from gene expression data (using the DoRothEA regulon^7^ together with the VIPER algorithm^8^), and pre-processing and filtering TFs and samples, 3) constructing a prior knowledge network (PKN) to act as a scaffold for the deep learning model, 4) train cell line-specific models, 5) extracting the inferred DTIs from the model, 6) estimating off-target effects of drugs on specific TFs and selecting a sample of interest, and 7) ultimately extract the mechanism of action (MoA) of a drug’s off-target effect on a TF, in the form of a smaller signaling network.

As an example of these steps, we use transcriptomic data from the L1000 dataset^9^ to train an ensemble of 50 models simulating drug-induced transcriptional responses on the A375 cancer cell line. Then, after extracting learned DTIs, we build a signaling network explaining Lestaurtinib’s off-target effects on the FOXM1 transcription factor. All steps can also be found in the GitHub repository: https://github.com/Lauffenburger-Lab/DrugsANNSignaling.

There are several files that are generated and used in the protocol. Here we describe the most important ones that are required for training a model and using it for a case study. “***DrugsIn”*** is the input file (X) of the model in .tsv format, containing the input drug concentrations, with samples in the rows and the available drugs in the columns. This is manually curated by the user. ***“TargetsIn”*** is a .tsv file containing prior knowledge of drug-target interactions with drugs in the rows and targets in the columns. This is generated in **Step 1**. ***“TFsOut”*** is the output file (Y) of the model in .tsv file format, containing output TF activities. This is generated in **Step 2**. ***“PKN”*** is the prior knowledge signaling network which will be used as a scaffold for the neural network model. This is generated in **Step 3**, or you can provide a manually curated one. ***“PknAnnotation”*** is the Annotation file for the PKN, generated in **Step 3**, or you can provide a manually curated one. ***“ChemicalSims”*** is a .csv file containing chemical similarities between drugs in a matrix format. This is generated in **Step 4**.

### Retrieve drug-target interactions

**Timing: <30 s**

Generally, for your own study, you need to:

1. Retrieve drug-target interactions from a database, e.g., DrugBank,^10^ Broad’s Institute Repurposing Hub.^11^
2. Select the drugs that you use in your study.
3. Find which drugs’ targets that overlap with your prior knowledge network.
4. Use this to construct a DTI matrix, where rows are drugs and columns are targets.
  In this example tutorial
  a. In the GitHub repository:
    i. Go to the preprocessing/preprocessed_data/TrainingValidationData folder.
    ii. Find the “***L1000_lvl3_allcells-drugs_targets_A375.tsv***” file that contains the available DTIs for the case study of using the approach for training models for the A375 cell line. You will need this file in the subsequent steps.
      ***Note:*** You may use your own drug-target space, but make sure that the data are saved in the same format (i.e. binary matrix in a TSV file with drugs’ SMILES set as the names of the rows and targets’ UniProt IDs set as the names of the columns), namely: 1) a .tsv file, 2) the names of the rows are saved and are the available drugs, 3) the names of the columns are saved and are the names of the available targets.

### Infer transcription factor activity

**Timing: 2–30 min**

This step is not necessary when using pre-compiled data from the original publication.^1^ The TF activities are already inferred and pre-compiled in the “***TrimmedFinal_l1000_allgenes_lvl3_tfs.tsv***” file in the preprocessing/ preprocessed_data/ TrainingValidationData folder. Here, it is described how to infer TF activities from gene expression data, in case a user wants to use their own transcriptomic dataset or augment the data already available in the L1000 dataset, which was used in this study.

***Note:*** The timing of this step is proportional to the number of samples you use. For example, for ∼10 000 samples with ∼10k genes measured it takes ∼2 min but for the ∼164 000 samples used from the level 3 data of the L1000 dataset it takes ∼32 min.

**Figure 1:**
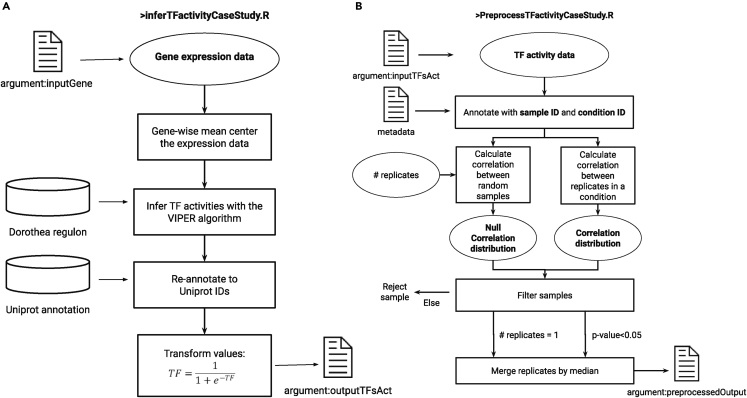
Schematic description of the information flow of the algorithms used to (**A)** infer transcription factor activity from gene expression data, and (**B)** filter out conditions where the correlation between replicates is not higher than random

A schematic explanation of the information flow for the algorithms of “***inferTFactivityCaseStudy.R”*** (used to infer TF activity) is available in Figure 1A.

5. Open Rstudio (or a terminal where you can execute R scripts) and make sure the working directory is the preprocessing folder.
  a. You can click on the preprocessing.Rproj file and this will start RStudio in the appropriate folder.
  b. > getwd() can show the current working directory.
  c. > setwd(dir) can change the current working directory to `dir`, which is the folder path of your choice.
6. Go to the `terminal` (not the console) section of RStudio and run the command:

> Rscript inferTFactivityCaseStudy.R [inputGene] [outputTFsAct]

***Note: [inputGene]*** is a file saved in .rds format containing the gene expression (you can convert your CSV or TSV files using the CSV_TSV_to_RDS.R script located in the preprocessing folder), with gene names (as gene symbols) being at the rows and sample names at the columns. For the case of the L1000 dataset, you can use as input the following path: '***preprocessed_data/l1000_all_genes_lvl3_drugs_with_targets_exemplar.rds***'. ***[outputTFsAct]*** is the file where the inferred TF activities will be saved in .rds format. The TF names are in the columns and sample names are now in the rows.

***Note:*** The ‘inputGeneExpr’ is the variable in the script that takes the value of the [inputGene] argument, and it contains the gene expression saved in .rds format in this case study. In case you have saved your gene expression in a different format use the appropriate command to read the file in line 13 (or you may convert your CSV or TSV files using the CSV_TSV_to_RDS.R script located in the preprocessing folder). Additionally, you may run everything line by line in RStudio but then you need to specify the input and output files in lines 10–11.

***Note:*** VIPER^8^ which is used to infer transcription factors (TFs) activities outputs a normalized enrichment score (following a normal-like distribution) describing the activity of the TFs. Our framework requires TF activity to be within a range of zero to one, thus a sigmoid transformation is applied: TF_activities <- 1/(1+exp(-TF_activities)).

**CRITICAL:** In case you get a memory error, see Troubleshooting 1.

**CRITICAL:** The genes should be annotated with gene symbols (using the NCBI standard) throughout the whole tutorial and analysis.

### Data filtering

**Timing: ∼1 h for the L1000 dataset**

This step is optional, but in case you want to filter the data to only keep high-quality samples and TFs, we created a process (Figure 1B) for filtering out samples based on poor correlation between technical replicates within the sample. To determine if the correlation between replicates is statistically significant, we build a null distribution of random correlations between TF activity profiles. More details on this process are discussed in the original publication by Meimetis et al.^1^

7. Open Rstudio (or a terminal where you can execute R scripts) and make sure the working directory is the preprocessing folder.
8. Go to the `terminal` (not the console) section of RStudio and run the command:

>RscriptPreprocessTFactivityCaseStudy.R[inputTFsAct][preprocessedOutput]

***Note: [inputTFsAct]*** is the file where the inferred TF activities are saved in .rds format. The TF names are in the columns and the sample names at the rows. ***[preprocessedOutput]*** is the file where the preprocessed and filtered TF activities will be saved in. tsv format. The TF names are in the columns and sample names are in the rows.

**CRITICAL:** The whole script uses specific identifiers and data structures of the L1000 dataset, especially lines 20–37. For example, it calculates the correlation between replicates and aggregates them by assuming that each replicate is identified by a unique `inst_id`, while each condition consisting of multiple replicates is identified by a unique `sig_id`. These are identifiers unique to the L1000 and they are called and used by the functions. Other L1000-specific variables are also called in this script. Several core ideas from this optional preprocessing step could in principle be applied to other datasets, such as comparisons of intra-condition correlations with null distributions, but in general, preprocessing is dependent on the structure and design choices of the researchers that generated the dataset. Always what is required is some kind of sample ID (e.g. `inst_id` in the L1000) and condition ID (e.g. `sig_id` in the L1000).

***Note:*** We suggest running preprocessing in a computer cluster, otherwise you might get a memory error (see Troubleshooting 1) or it will take a lot of time without enough computing power.

### Extract a prior knowledge signaling network

**Timing: ∼5 min**

**Figure 2:**
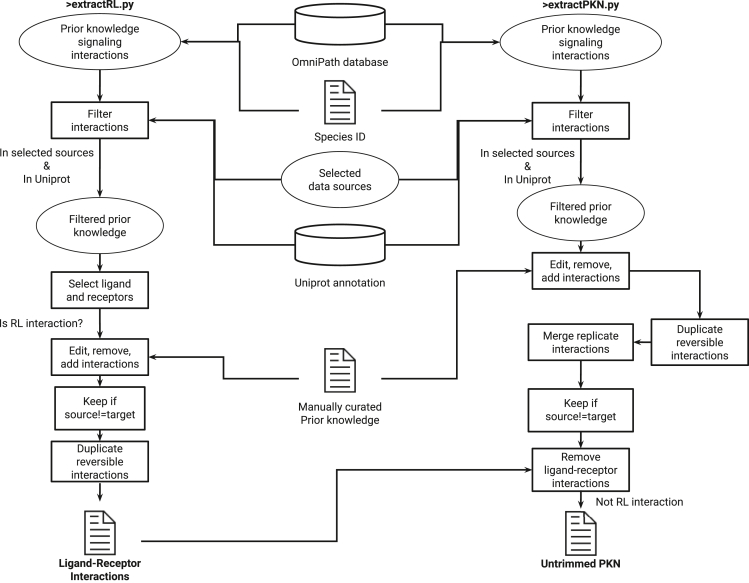
Schematic description of the information flow of the algorithms used to extract a prior knowledge network (PKN) and receptor-ligand interactions

**Figure 3:**
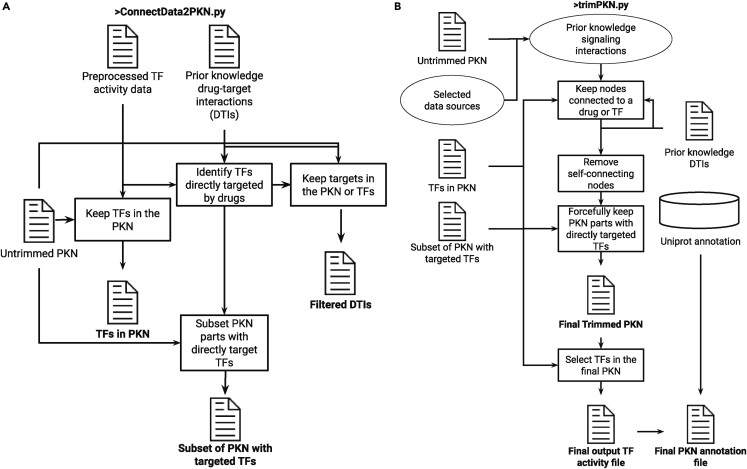
Schematic description of the information flow of the algorithms used to generate and trim the (**A)** prior drug-target interactions and (**B)** the final PKN, that will be used as the scaffold for the signaling part of the modeling

In this step, you will generate prior knowledge networks (PKNs) that will serve as the scaffolds for your model. We provide scripts for extracting a network from the OmniPath^12^ resource, but you may use your own network following the same syntax. Starting with ligand-receptor interactions, you will extract, curate (Figure 2), and trim (Figure 3) the networks, then similarly you will curate and trim a prior knowledge signaling network of signaling. **If you want to use the same networks as Meimetis** et al. **used in their study****^1^****, you may skip this step and use the files that have already been generated in GitHub**.

9. Visit the web page of OmniPath and retrieve the latest version (in the tutorial this is already retrieved and saved in the data folder): https://archive.omnipathdb.org. In this tutorial this is saved as omnipath_webservice_interactions__recent.tsv in the data folder.
10. Open a terminal where you can run Python scripts and navigate to the preprocessing folder.
11. Extract Ligand-Receptor interactions from OmniPath^12^ and automatically save the result:

> python ./extractRL.py --RLFull file1.tsv --RL file2.tsv --species 9606 --WholePKN file3.tsv--add_curation file4.tsv --remove_curation file5.tsv --edit_curation file6.tsv

***Note:*** “species” denotes the species id, with default = 9606, which is the id for human interactions. “RLFull” denotes all receptor-ligand interactions in. tsv format (default = preprocessed_data/PKN/RLFull.tsv). “add_curation” are the interactions to manually add (default = preprocessed_data/RL/add.tsv).“remove_curation” are the interactions to manually remove (default = preprocessed_data/RL/remove.tsv). “edit_curation” are the interactions to manually edit (default = preprocessed_data/RL/edit.tsv). “RL” are the receptors-ligands in .tsv format filtered (default = preprocessed_data/PKN/RL.tsv). “WholePKN” denotes the whole retrieved prior knowledge network of signaling that is used to construct the PKN that will be used in the model (default = ../data/omnipath_webservice_interactions__recent.tsv).

***Note:*** You can keep all the default arguments and manually edit the add.tsv, remove.tsv, and edit.tsv files (which are initially empty) **in the RL folder** to curate some receptor-ligand interactions.

***Note:*** In line 24 we keep interactions only from specific sources in the OmniPath database.^12^ If you want to change that you need to modify these lines.

12. Extract the raw network from OmniPath^12^ and automatically save the result:

> python ./extractPKN.py --species_id file1.tsv --add_curation file2.tsv --remove_curation file3.tsv --edit_curation file4.tsv --pknFull file5.tsv --pknUniprot file6.tsv --RLinteractions file7.tsv --WholePKN file8.tsv

***Note:*** “species_id” denotes the species id, with default = 9606, which is the id for human interactions. “add_curation” are the interactions to manually add (default = preprocessed_data/PKN/add.tsv).“remove_curation” are the interactions to manually remove (default = preprocessed_data/PKN/remove.tsv). “edit_curation” are the interactions to manually edit (default = preprocessed_data/PKN/edit.tsv). “RLinteractions” are the receptors-ligands in .tsv format filtered (default = preprocessed_data/PKN/RL.tsv). “pknFull” are all the kept interactions before trimming in .tsv format (default = preprocessed_data/PKN/pknFull.tsv). “pknUniprot” are all the kept interactions with UniProt IDs in .tsv format (default = preprocessed_data/PKN/pkn.tsv). “WholePKN” is the whole retrieved prior knowledge network of signaling that is used to construct the PKN that will be used in the model (default = ../data/omnipath_webservice_interactions__recent.tsv).

***Note:*** You can keep all the default arguments and manually change the add.tsv, remove.tsv, and edit.tsv files (which are initially empty) **in the PKN folder** to curate some interactions.

***Note:*** In line 39 we keep interactions only from specific sources in the OmniPath database.^12^ If you want to change that you need to modify these lines.

13. Keep TFs, drugs, and targets that can be connected to the PKN, and save parts of the PKN to forcefully keep even after trimming (Figure 3A):

> python ./ConnectData2PKN.py --pknPath pnkFile.tsv --DTIpath DTIFile.tsv --Tfpath input1.tsv --targetedTFs output1.tsv --forced2keep output2.tsv --outTrimmedTFs output3.tsv --outTrimmedDTIs output4.tsv

***Note:*** “pknPath” is the untrimmed PKN file in .tsv format (default = preprocessed_data/PKN/pkn.tsv). “DTIpath” is the untrimmed drug-target interactions in long format saved in .tsv format (default = preprocessed_data/PKN/L1000_lvl3_DT.tsv). “TFpath” is the untrimmed TF activity file in .tsv format. “targetedTFs” is the path to save TFs that are directly targeted by a drug in .tsv format. “forced2keep” is the path to save PKN parts forcefully kept because they contain targeted TFs. “outTrimmedTFs” is the path to re-save TF activities, trimmed to include only TFs in the PKN. “outTrimmedDTIs” is the path to re-save DTIs, trimmed to include only drugs with targets in the PKN.

14. Reduce the network to contain only interactions from specific sources, trim disconnected parts and dead-ends, keep drug/targets and TFs that are in the network, and automatically save the result (Figure 3B):

> python ./trimPKN.py --coreSources s1 s2 s3 s4 --pknUniprot file1.tsv --RLinteractions file2.tsv --DTIpath file3.tsv --TFpath file4.tsv --targetedTFs file5.tsv --forced2keep file6.tsv --TrimmedTFpath outFile1.tsv --FinalPKN outFile2.tsv --FinalPKNAnnotation outFile3.tsv

***Note:*** “pknUniprot” are all the kept interactions with Uniprot ids in .tsv format (default = preprocessed_data/PKN/pkn.tsv). “RLinteractions” are the receptors-ligands in .tsv format filtered (default = preprocessed_data/PKN/RL.tsv).“DTIpath” are the trimmed drug-target interactions in long format saved in .tsv format (default = preprocessed_data/PKN/L1000_lvl3_DT.tsv). “targetedTFs” is the path to load TFs that are directly targeted by a drug in .tsv format (default = preprocessed_data/TF_activities/tfs_targetd_alls_genes_lvl3.tsv). “forced2keep” is the path to load PKN parts to forcefully keep because it contains targeted TFs (default = preprocessed_data/PKN/L1000_latest_Add_lvl3.tsv). “TFpath” are the input TF activities to be finally trimmed. “TrimmedTFpath” is the final trimmed TF activity file in .tsv format. “FinalPKN” is the final, trimmed PKN file in .tsv format. “FinalPKNAnnotation” is the final, trimmed annotation file for the PKN in .tsv format.

**CRITICAL:** The drug-target interaction files used here are not the files used to train the model. That file needs to be in long format (not matrix format) and has been modified to include control conditions (such as DMSO, PBS, etc.). You may use the script located inside the preprocessing folder (MatrixLongFormatConversion.py) to convert your drug-target interaction file from matrix to long format, and vice versa.

**Figure 4:**
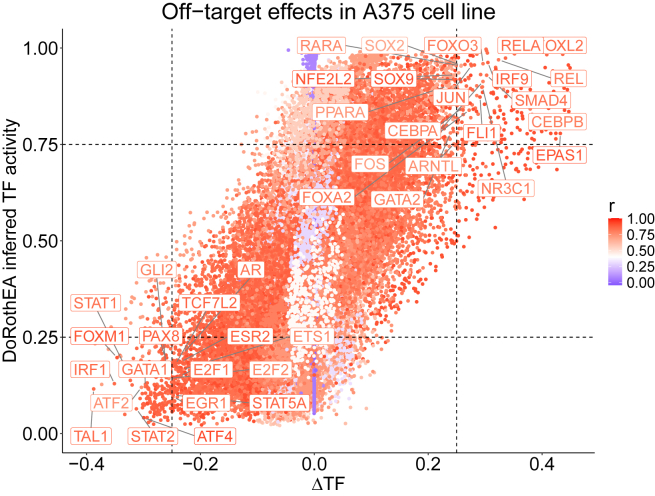
The calculated activity of the TFs compared with the predicted off-target effects alongside a confidence score from derived from the ensemble performance in training for each TF Each point corresponds to a specific drug-TF pair.

### Train cell-line-specific models

**Timing: ∼6.5 h per model**

In this step, you will train models to predict the TF activity of drug perturbations in a specific cell line, utilizing in the process the chemical similarity between drugs and their known targets, in a pre-defined drug-target space (pseudo-code in Table 1).

**CRITICAL:** Before beginning it is essential to calculate all chemical similarities and have obtained all necessary input files, either manually or via the previous steps, 5–6 files:

**Table 1:** Pseudo-code for training a DT-LEMBAS model corresponding to the DTLembasTrainOneCaseStudy.py script

**Input:** ➢ $X_{i}$, [bs x d] matrix for bs samples (same as the batch size bs) and d available drugs with each drug’s concentration for a given sample. ➢ drugLayer, the layer of DT-LEMBAS that generates perturbation signal on potential targets, given drugs’ concentrations. ➢ LEMBAS, the signaling layer of DT-LEMBAS that takes as input a signal matrix${Y_{in}}_{i}$[bs x t], where t is the number of available targets, and predicts the activity of signaling nodes (${Yfull}_{i}$) and transcription factors ($Y_{i}$). **Output:** ➢ ${Yfull}_{i}$, [bs x n] matrix for bs samples (same as the batch size bs) and n dimensions for the number of signaling nodes, containing the activity of signaling nodes. ➢ $Y_{i}$, [bs x m] matrix for bs samples and m TFs containing the predicted TF activity. ➢ loss, the value of the objective function used to train the model. ➢ Updated weights for all parts of the model. **for** epochs **steps:** **for** batches **steps:** X,Y←get data for the current batch ${\;Y}_{in}\;\leftarrow \;drugLayer\left(X\right)$*# calculate perturbation signal* $Y_{in}\;\leftarrow \;position\;correctly\;Y_{in}\;in\;a\;vector\;with\;the\;size\;of\;all\;signaling\;nodes$ $Y_{in}\;\leftarrow \;Y_{in}+\lambda_{noise}*N\left(0,1\right)$ $\hat{Y},\hat{Y}full\leftarrow LEMBAS\left(Y_{in}\right)$$error\leftarrow mean\left({\left(Y-\hat{Y}\right)}^{2}\right)$ loss←error+SignalingRegularization+DrugLayerRegularization calculategradientswithbackpropagation updateweights

For a detailed description of the regularization terms, refer to the Meimetis et al. publication.^1^

 DrugsIn: A .tsv file containing the input drug concentration, with samples in the rows and the pre-defined drugs in the columns.

 TargetsIn: A .tsv file containing prior knowledge of drug-target interactions with drugs in the rows and targets in the columns

 TFsOut: A .tsv file containing output TF activities, with samples in the rows and TFs in the columns

 ChemicalSims: A .csv file containing chemical similarities between drugs in a matrix format with the smiles of the drugs being the columns’ and rows’ names. This file should be located in the ChemicalSims folder in the preprocessing folder of the GitHub repository, and instructions on creating are given below.

 PKN: The prior knowledge signaling network which will be used as a scaffold for the neural network model. This should have been generated in the previous steps, or you can provide a manually curated one.

 PknAnnotation: Annotation file for the PKN, also generated in the previous step.

To calculate chemical similarities:

15. Open a terminal where you can run Python and go to the ChemicalSims folder in the preprocessing folder of the GitHub repository.
16. Create a .csv file with one column named ‘smiles’ containing the SMILES of the drugs for which you want to calculate pairwise chemical similarities and save it in the ChemicalSims folder.
17. Run python ./**smiles_similarity_ecfp4.py** and follow the instructions that will be presented on your screen.

If all the requirements are satisfied you are ready to train a model.

18. Open a terminal where you can run Python and go to the learning folder of the GitHub repository.
19. Run the command:

>python ./ DTLembasTrainOneCaseStudy.py --DrugsIn file1 --TargetsIn file2 --TFsOut file3 --ChemicalSims file4 --PKN file5 --PknAnnotation file6 --res_dir folder --outPattern modelCellLineX --no 1

***Note:*** The ***“DrugsIn”***, ***“TargetsIn”***, ***“TFsOut”***, ***“ChemicalSims”***, ***“PKN”***, and ***“PknAnnotation”*** arguments have been explained above.***“res_dir”*** should be a folder path to save trained models and output some figures of the training performance, loss, and other model diagnostics. ***“outPattern”*** is the pattern/name (without the file extension and model number) that will be used when saving multiple models and figures for this specific case study. ***“no”*** is an integer number serving as an identifier to denote which one of the multiple models of the ensemble is currently being trained.

20. After training **at least 20 models (we have run 50 for 33 cell lines)**, to later use as an ensemble of neural networks to make predictions, run the command:

**>python ./CellLineSpecificEvalEnsembleALL.py, with the correct arguments** (these arguments correspond to file paths and parameters such as the number of models in the ensemble) to evaluate how well the model fits the training data. The performance is stored in ***[CellPrefix]_trainPerformance_perTF.csv*** at a designated results’ directory.

***Note:*** There is another argument available which is --***model_type*** and the default value is 4. This was used to test a couple of different variations of the drug module in the original study.^1^ The best one that was further used was denoted as model_type 4, and thus it is suggested to keep this always with the default value.

***Note:*** Well-fitted TFs with a high chance of generalization correspond to the top 25% of TFs in terms of performance (more than ∼0.4 Pearson correlation across models and cell lines) and poorly-fitted TFs whose predictions should not be trusted correspond to less than ∼0.2 correlation in training.^1^

***Note:*** Our approach requires training an ensemble of multiple models. The training script can be submitted as a batch job using a job scheduler (**e.g. using Slurm**) to a single-threaded computer cluster of CPU nodes, enabling this way the parallelized training of multiple models. To achieve this, the user needs to create a shell script containing commands involved in executing the job. For example, a script named **train.sh** be created with the following lines inside of it (comments inside the parentheses should be deleted):

#!/bin/bash

#SBATCH -N 1 (number of nodes to use)

#SBATCH -n 16 (number of CPUs per node to use)

#SBATCH --mail-type=ALL (sent email notifications regarding the job)

#SBATCH --mail-user=user@mit.edu (email for notification to be sent to)

#SBATCH --array=0-49 (array of jobs submitted, equal the number of models)

#############################################

# Load module

module load miniconda3/v4

source /home/software/conda/miniconda3/bin/condainit

conda activate ann

python3 ./DTLembasTrainOneCaseStudy.py --DrugsIn ′../data/L1000_lvl3_A375-conditions_drugs.tsv′--TargetsIn ′../data/L1000_lvl3_ A375-drugs_targets.tsv′ --TFsOut ′../data/TrimmedFinal_l1000_allgenes_lvl3_tfs.tsv′ --ChemicalSims ′../preprocessing/preprocessed_data/ChemicalSims/lvl3_similarities_ A375.csv′ --res_dir ′case_study′ --outPattern "l1000_latest_model_modeltype4_ A375_case_study" --model_type 4 --no $SLURM_ARRAY_TASK_ID

Then to submit the jobs, a user can run:

> sbatch train.sh

### Extract inferred drug-target interactions

**Timing: ∼4 min per model**

In this step, you will extract the inferred DTIs that can explain the TF activity output, that the model learned through training (pseudo-code in Table 2).

21. Navigate to the MoA folder of the GitHub repository and open a terminal where you can run Python.
22. Run the following command to extract drug-target interaction scores (using integrated gradients^4^):

> python ./inferEnsembleScoreCaseStudy.py --ensembles_path foler1 --inputPattern modelCellLineX --numberOfModels 50 --drugInputFile file1 --drugTargetsFile file2 --TFOutFile file3 --drugSimilarityFile file4 --interactionsPath folder2 --Y_ALL_path folder3/Y_ALL.pt --Y_ALL_masked_path folder3/Y_ALL_masked.pt --ig_n_steps 10

***Note: “ensembles_path”*** should be the path to the ensemble of saved models. ***“inputPattern”*** The pattern/name (without the file extension and model number) that was used when saving multiple models for this specific case study. ***“numberOfModels”*** is the number of models that were trained in the ensemble (default = 50). ***“drugInputFile”*** is the same .tsv file that was used for training the models, containing the input drug concentration, with samples in the rows and the pre-defined drugs in the columns. ***“drugTargetsFile”*** is the same .tsv file that was used for training the models, containing prior knowledge of drug-target interactions with drugs in the rows and targets in the columns. ***“TFOutFile”*** is the same .tsv file that was used for training the models, containing output TF activities, with samples in the rows and TFs in the columns. ***“drugSimilarityFile”*** is the same .csv file that was used for training the models, containing chemical similarities between drugs in a matrix format with the smiles of the drugs being the columns’ and rows’ names. ***“interactionsPath”*** should be the path of the folder to save .csv files containing drug-target interaction scores for every model. ***“Y_ALL_path”*** is the full path and filename, to save a .pt (pytorch tensor) file, containing predicted values of training data by the models. It has dimensions [numberOfModels , # samples, # TFs] . ***“Y_ALL_masked_path”*** is the full path and filename, to save a .pt (pytorch tensor) file, containing predicted values of training data when masking out potential interactions based on their integrated gradient score and specified threshold to binarize interactions. The dimensions of this tensor are [numberOfModels, # thresholds, # samples, # TFs]. ***“ig_n_steps”*** is the number of points to be used in the integral when using the integrated gradients approach (default = 10, **should not be less than 10**).

***Note:*** The score thresholds tested are 50 and range from 10^-3.5^ to 10^3.5^ in a logarithmic space. These same thresholds are used again in the next step of extracting drug-target interactions. If you experience issues with the speed of this process, or perhaps you want higher granularity in the calculated error later check Troubleshooting 2.

23. Run the following command to ultimately extract binary drug-target interaction and the threshold for inferring them **for each drug,** which corresponds to an average error increase of 25% in predicting TF activity for each drug:

> python ./InferDTICaseStudy.py --ensembles_path foler1 --inputPattern modelCellLineX --numberOfModels 50 --drugInputFile file1 --drugTargetsFile file2 --TFOutFile file3 --drugSimilarityFile file4 --Y_ALL_path folder3/Y_ALL.pt --Y_ALL_masked_path folder3/Y_ALL_masked.pt --interactionsPath folder2 --error_threshold 0.25

***Note:*** All the arguments with the same name as before have already been explained, and you can use the same again.

***Note:* “--*error_threshold***”: The maximum allowable average error increase across all TFs when masking out interactions, which leads to identifying the appropriate interaction score threshold for each drug.

***Note:*** This script saves the thresholds to consider interactions for each drug and all inferred binary interactions in the folder of ensembles_path.

***Note:*** Similar to the Meimetis et al.^1^ publication, to benchmark the inference of drug-target interactions (DTIs) you may use an external drug-target interaction dataset, where DTI inference is treated as a binary classification problem. This means they may use accuracy and F1 score to evaluate the performance in inferring DTIs, as well as to tune different thresholds selected for inferring interactions. However, since in this formulation of the problem the true negatives (no interaction between drug and target) are not really well known, we recommend the user to also inspect the rate of discovering new interactions, since for studying off-target effects it is expected to infer new interactions and not only reproducing prior knowledge. More details and extensive benchmarking can be found in the original publication.^1^

**CRITICAL:** In case you have multiple control conditions or generally duplicate conditions the script calculates a threshold for each one of the multiple instances of the perturbation returns the average threshold. In this case study (as well as the original work by Meimetis et al.^1^) this happens only in the case of DMSO. This script however will not work in the case of drug combinations, and generally, the approach has not been benchmarked for the case of drug combinations at all.

**CRITICAL:** The inferEnsembleScoreCaseStudy.py will automatically utilize a GPU if available to calculate the drug-target interaction scores. **The timing denoted here is for the GPU implementation. In the absence of a GPU**, for this specific drug module which is also linear, the user can calculate the same score by performing all appropriate linear algebra calculations (pseudo-code in Table 3) by running:

> python ./ InferInteractionScoresLinAlg.py --ensembles_path foler1 --inputPattern modelCellLineX --numberOfModels 50 --drugInputFile file1 --drugTargetsFile file2 --TFOutFile file3 --drugSimilarityFile file4 --interactionsPath folder2 --Y_ALL_path folder3/Y_ALL.pt --Y_ALL_masked_path folder3/Y_ALL_masked.pt.

**Table 2:** Pseudo-code for extracting inferred drug-target interactions from DT-LEMBAS, using integrated gradients, corresponding to inferEnsembleScoreCaseStudy.py and InferDTICaseStudy.py

**Input:** ➢ Input drug concentrationsX, [N x d] matrix with N samples and d available drugs with each drug’s concentration for the a given sample. ➢ model, a whole DT-LEMBAS model, with pseudo-classed drugLayer denoting, only the drug layer part of DT-LEMBAS, and LEMBAS denoting the signaling layer part. ➢ thresholds, thresholds used to binarize the drug-target interaction scores. **Output:** ➢ $\hat{Y}$[N x m], predicted activity for each of m TFs. ➢ ${\hat{Y}}_{masked}$[N x m], predicted activity for each of m TFs, when masking drug-target interactions for different thresholds. ➢ scores, the drug-target interaction scores. ➢ binary drug-target interactions based on a selected threshold. ➢ global_thresholds, the interaction score used to extract interactions for each drug for each model **Symbols:** ➢ ·→matrix multiplication ➢ ⊙→element-wise matrix multiplication **>inferEnsembleScoreCaseStudy.py:** **for**i**steps in range(# models):** ${\hat{Y}}_{i}\leftarrow model_{i}\left(X\right)$ $scores_{i}\;\leftarrow IntegratedGradients\left(model_{i},X\right)$#using the Captum library **for** j **steps in range(# thresholds):** $T\leftarrow absolute\left(scores\right)\leq thresholds_{j}$ mask←T·(X≠0) $X_{in}\leftarrow model.drugLayer\left(X\right)$ ${Y_{masked}}_{ij}\leftarrow \mathrm{mod}\;el.LEMBAS\left(X_{in}\right)$ **>InferDTICaseStudy.py:** **for**i**steps in range(# models):** ***for****k****steps in range(# drugs):*** $err_{1}\leftarrow \frac{1}{\#\;TFs}\sum_{TF}\left(absolute\left(Y_{masked_{ik}}-Y_{ik}\right)\right)$# vector with error values for each threshold $err_{0}\leftarrow \frac{1}{\#\;TFs}\sum_{TF}\left(absolute\left(Y_{ik}-Y_{k}^{true}\right)\right)$# scalar $percentage\;error\;\leftarrow \frac{err_{1}-err_{0}}{err_{1}\left[1\right]-err_{0}}$ $global_{thresholds_{k}}\leftarrow average\;of\;thresholds$ wherethepercentageerrorbecomes0.25Binaryinteractions←scores≤identifiedthresholdforeachdrug

**Table 3:** Pseudo-code for extracting inferred drug-target interactions from DT-LEMBAS, for the case of using a linear layer, corresponding to the InferInteractionScoresLinAlg.py script

Run instead of integrated gradients when using the published drug layer
**Input:** ➢ S, [drugs x drugs] matrix containing pre-calculated chemical similarity of drugs. ➢ $W_{drug}$, [drugs x drugs] matrix containing trained weights to scale chemical similarity. ➢ A, [targets x drugs] matrix containing trainable weights for drug-target interactions. ➢ mask, [targets x drugs] binary mask of known drug-target interactions. ➢ $w_{bn}$, [drugs] vector with trained weights of the batch normalization layer in the drug layer. ➢ ${var}_{bn}$, [drugs] vector with trained running variances of the batch normalization layer in the drug layer. ➢ ε, scalar used in the batch normalization of the drug layer (by default: 10^-5^) **Output:** ➢ scores, [drugs x targets] matrix containing all extracted drug-target interaction scores. **Symbols:** ➢ ·→matrix multiplication ➢⊙→element-wise matrix multiplication $W=S⊙W_{drug}$ A=A⊙mask $K=\frac{w_{bn}}{\sqrt{var_{bn}+\epsilon }}$ $scores={\left(A\cdot {\left(K⊙W\right)}^{T}\right)}^{T}$

This script takes approximately the same time (∼2.5 min per model) requiring only CPU to run.

**CRITICAL:** The inferEnsembleScoreCaseStudy.py utilizes integrated gradients to calculate the drug-target interaction scores, with 10 integration steps. However, the steps are only 10 because of the linear nature of the drug module. For more complex non-linear modules, the user should experiment with a larger number of steps (100 or 1000 steps, until convergence is observed).

### Identify off-target effects on transcription factors

**Timing: ∼15 s per model**

In this step, you will estimate the off-target effects of all drugs on the activities of each of the TFs, and save this off-target effect estimate in the file DeltaTF1.csv (pseudo-code in Table 4). The main point is to ultimately identify a drug-TF pair of interest, where the drug significantly affects the TF’s activity because of off-target effects. Additionally, as a performance metric for each TF, the average fitted TF across all models in the ensemble is compared with the true value in terms of its Pearson correlation. This performance metric will be saved in the TrainEnsemblePerformance.csv file, in the same folder (res_dir, see below) as DeltaTF1.csv. The figure and data frame combining these files to identify a sample of interest are generated in Step 6.4.

24. Open a terminal where you can run Python and navigate to the postprocessing folder of the GitHub repository.
25. Run the command:

> python ./inferOffTargetEffectDeltaCaseStudy.py –inputPathPattern folder/modelCellLineX --DrugsIn file1 --TargetsIn file2 --TFsOut file3 --ChemicalSims file4 --PKN file5 --PknAnnotation file6 --res_dir folder --numberOfModels 50

***Note: “inputPathPattern”*** is the full folder path and file name pattern (without the file extension and model number) to be used to load trained models. ***“DrugsIn”***, as in the training step, it is the .tsv file containing the input drug concentration, with samples in the rows and the pre-defined drugs in the columns. ***“TargetsIn”***, as in the training step, it is the .tsv file containing prior knowledge of drug-target interactions with drugs in the rows and targets in the columns. ***“TFsOut”***, as in the training step, it is the .tsv file containing output TF activities, with samples in the rows and TFs in the columns. ***“ChemicalSims”***, as in the training step, it is the .csv file containing chemical similarities between drugs in a matrix format with the smiles of the drugs being the columns’ and rows’ names. ***“PKN”*** is the prior knowledge signaling network which will be used as a scaffold for the neural network model. This should have been generated in the previous steps, or you can provide a manually curated one. ***“PknAnnotation”*** is the annotation file for the PKN, also generated in the previous steps. ***“res_dir”*** is the Folder path to save the off-target effect estimates and the training ensemble performance of the models. ***“numberOfModels”*** is used in the ensemble approach (default = 50).

26. Open Rstudio (or a terminal where you can execute R scripts) and make sure the working directory is the postprocessing folder.
  a. You can click on the postprocessing.Rproj file and this will start Rstudio in the appropriate folder.
  b. > getwd() can show the current working directory.
  c. > setwd(dir) can change the current working directory to `dir`, which is the folder path of your choice.
27. Open the **chooseTFsWithOffTargetsCaseStudy.R** in Rstudio (**or run the command** **> Rscript chooseTFsWithOffTargetsCaseStudy.R** **in a terminal**):

***Note:*** You can run line-by-line interactively to interrogate the results.

***Note:*** This script will show and then save a figure (Figure 4) visualizing the off-target effect and activity of each TF under each drug perturbation, as well as the performance in predicting it correctly.

***Note:*** Additionally, it saves a file with the maximum performance and off-target for each TF to later examine interesting case studies.

***Note:* Importantly, you may interrogate the xlsx file exported at the path present in line 29 in the script, to manually identify more case studies based on your choices regarding when there is a large off-target effect (based on the absolute value of the `delta` column variable), when a TF is well-fitted (based on the `r` column variable), and the induced activity of the TF (the `activity` column variable). In the saved .csv file with interesting samples only the maximum off-targets are saved.**

**CRITICAL:** First, change and use the appropriate file and folder paths for saving and loading files, for your case study.

***Note:*** In this study, we denote as large estimated off-target effects those with an absolute value ≥ 0.25 (or relaxed to ≥0.2). Meanwhile, the user should perhaps investigate the cases that also the actual induced TF activity is more than 0.75 or less than 0.25 since this means that the TF is active or inactive, respectively, because of the off-target effects.

**Table 4:** Pseudo-code estimating the off-target effects of each drug in the data on each available transcription factor, corresponding to the inferOffTargetEffectDeltaCaseStudy.py script

**Input:** ➢ Input drug concentrationsX, [N x d] matrix with N samples and d available drugs with each drug’s concentration for the a given sample. ➢ drugLayer, denoting, only the drug layer part of DT-LEMBAS. ➢ LEMBAS denoting the signaling layer part of DT-LEMBAS. ➢ mask, [targets x drugs] binary mask of known drug-target interactions. ➢ True drug induced TF activityY, [N x m] matrix with N samples and m TFs. **Output:** ➢ ΔY, the difference between predicted TF activities without off-targets and with off-targets, which will act as the proxy of the off-target effect on a TF. ➢ Pearson correlation between predicted TF activity from an ensemble of models and true TF activity. **for** i **steps in range(# models):** $X_{in}\leftarrow drugLayer_{i}\left(X\right)$ ${\hat{Y}}_{i}\leftarrow LEMBAS_{i}\left(X_{in}\right)$ ${X_{in}}_{masked}\leftarrow mask\cdot \left(X\neq 0\right)$ ${\hat{Y^{\prime }}}_{i}\leftarrow LEMBAS_{i}\left({X_{in}}_{masked}\right)$ $\hat{Y}\leftarrow mean\left(\hat{Y}\right)$ $\hat{Y^{\prime }}\leftarrow mean\left({\hat{Y}}^{\prime }\right)$ $\Delta Y=\hat{Y^{\prime }}-\hat{Y}$ Ensemble performance$\leftarrow correlation\left(\hat{Y},Y\right)$

**Figure 5:**
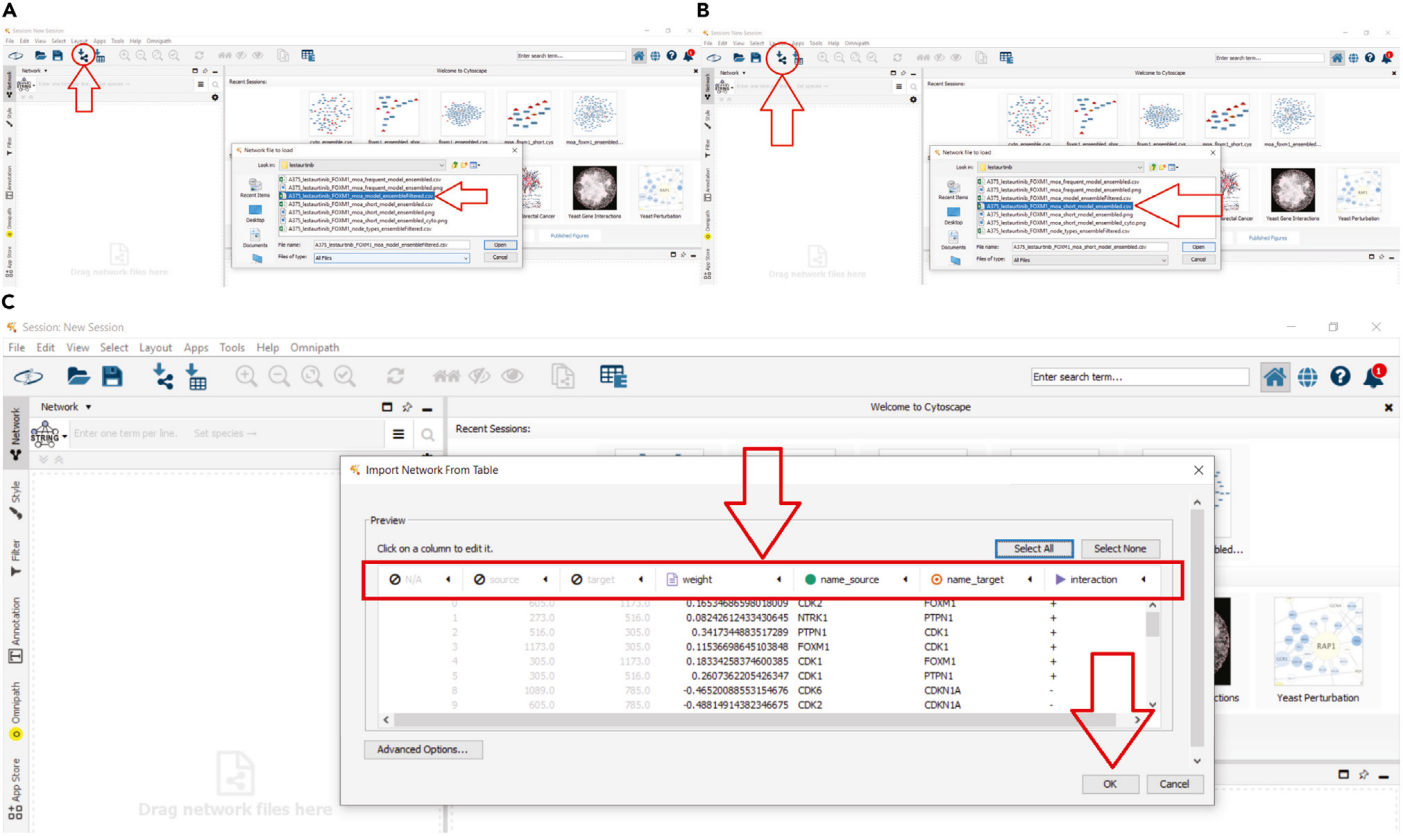
Examples of opening the extracted MoA network in the Cytoscape environment, together with the step-by-step actions taken by the user to open the results These are screenshots from Cytoscape. (**A)** Loading the network file for the ensembled reduced subnetwork. The red arrows show where the user needs to click to load the results. First, the user selects the “icon” to open a window where they can browse to find the files that contain the network (the second arrow). (**B)** Loading the shortest paths only for the ensembled reduced subnetwork. The red arrows have the same meaning as in panel A. (**C)** The columns and their assigned properties when loading the CSV file. The red arrows are added for emphasis to guide the user to the parts of the Cytoscape environment that they need to pay attention to (e.g., exactly which columns should be the source, target, and interaction properties of Cytoscape.

### Extract the mechanistic network explaining the off-target effect

**Timing: ∼1.5 min per model**

In this step, you will construct a mechanistic network explaining the off-target effect of a drug on a specific transcription factor. As an example, we conduct a case study where we investigate the off-target effects of the drug **Lestaurtinib** on the **FOXM1** transcription factor in the **A375** cell line.

**CRITICAL:** Before you begin, note that the following script requires you to have all previous results in a specific parent folder which contains the subfolders: i) models (containing the trained models), and ii) InteractionScores (where the drug-target interaction scores are saved). Additionally, this parent folder should contain the thresholds for inferring drug-target interaction in the all_drugs_global_thresholds.csv file. All of these files should already have been generated in the previous steps.

28. Open a terminal where you can run Python and navigate to the MoA folder.
29. Run the following command:

>python ./inferMoACaseStudy.py --inputPattern modelCellLineX --ensembles_path folder1--DrugsIn file1 --TargetsIn file2 --TFsOut file3 --ChemicalSims file4 --PKN file5 --PknAnnotation file6 --res_dir folder2 --interactionScorePattern interactionScores --numberOfModels 50 --source_freq_thresh 0.6 --edge_thresh_init 0.5 --moa_off_target "any" --Prefix "A375" --TF "Q08050" --TF_gene "FOXM1" --drug "C[C@]12O[C@H](C[C@]1(O)CO)n1c3ccccc3c3c4C(=O)NCc4c4c5ccccc5n2c4c13" --drug_name "lestaurtinib" --sample "CPC014_A375_6H:BRD-K23192422-001-01-1:10"

***Note: “inputPattern”*** is the pattern/name (without the file extension and model number) that was used when saving multiple models for this specific case study. ***“ensembles_path”*** should be the path to **the parent folder** which contains the `models` folder with the ensemble of saved models. ***“DrugsIn”***, as in the training step, it is the .tsv file containing the input drug concentration, with samples in the rows and the pre-defined drugs in the columns. ***“TargetsIn”***, as in the training step, it is the .tsv file containing prior knowledge of drug-target interactions with drugs in the rows and targets in the columns. ***“TFsOut”***, as in the training step, it is the .tsv file containing output TF activities, with samples in the rows and TFs in the columns. ***“ChemicalSims”***, as in the training step, it is the .csv file containing chemical similarities between drugs in a matrix format with the smiles of the drugs being the columns’ and rows’ names. ***“PKN”*** is the prior knowledge signaling network which will be used as a scaffold for the neural network model. This should have been generated in the previous steps, or you can provide a manually curated one. ***“PknAnnotation”*** is the Annotation file for the PKN, also generated in the previous steps. ***“res_dir”*** is the folder path to save results. ***“interactionScorePattern”*** is the pattern/name (without the file extension and model number) that was used when saving drug-target interaction scores for this specific case study. ***“numberOfModels”*** is the number of models that were trained in the ensemble (default = 50). ***“source_freq_thresh”*** is the minimum frequency score of a target being inferred by multiple models to finally consider it (default = 0.6). A stricter threshold can result in a smaller network, but may also result in an error because no targets are higher than the designated threshold. In such a case see Troubleshooting 3. ***“edge_thresh_init”*** is the initial minimum frequency score of an edge appearing in the signaling network when constructing the net explaining the MoA of the off-target (default = 0.5). **This is an initial filtering**, as subsequently this results in disconnecting all the drug’s targets and the TF of interest, edges are added to create a path connecting targets and TF with the highest possible sum of frequencies. ***“moa_off_target”*** is the sign of off-target effect to consider drug’s target. (default = "any", potential values ["any","inhibit","activate"]). **We strongly suggest keeping the default option. *“Prefix”*** is a name describing the biological system where the models were trained on (will be used when saving data). We recommend using the name of the cell line from which the data were derived. ***“TF”*** is the UniProt^13^ identifier of the transcription factor of interest. ***“TF_gene”*** is the gene name for the transcription factor of interest. ***“drug”*** is the SMILES of the drug of interest. The unique identifiers of drugs in the tutorial are SMILES and not commercial drug names, as the same chemical structure can have multiple commercial names. ***“drug_name”*** is the commercial (or any other) name of the drug of interest. ***“sample”*** is the Identifier of the perturbation used (in this case study we use the sig_id of the L1000 dataset, with more information available in the CLUE platform^14^ glossary.).

30. Run the following command to print the activity and interaction score of the target node, to investigate the sign of a drug-target interaction:

>python ./DrugTargetInteractionSignCaseStudy.py --inputPattern modelCellLineX --ensembles_path folder1--DrugsIn file1 --TargetsIn file2 --TFsOut file3 --ChemicalSims file4 --PKN file5 --PknAnnotation file6 --res_dir folder2 --interactionScorePattern interactionScores --numberOfModels 50 --moa_off_target "any" -- Prefix "A375" --node "P24941" --node_gene "CDK2" --drug "C[C@]12O[C@H](C[C@]1(O)CO)n1c3ccccc3c3c4C(=O)NCc4c4c5ccccc5n2c4c13" --drug_name "lestaurtinib" --sample "CPC014_A375_6H:BRD-K23192422-001-01-1:10"

***Note:*** The arguments are the same as the ones in the inferMoACaseStudy.py script, with the difference that instead of having as input a TF, the input is the name and UniProt identifier of any signaling node.

**CRITICAL:** All arguments in this script have the same values as those used in this case study.

31. Open Cytoscape^6^ and load the appropriate network (Figure 5).

**Figure 6:**
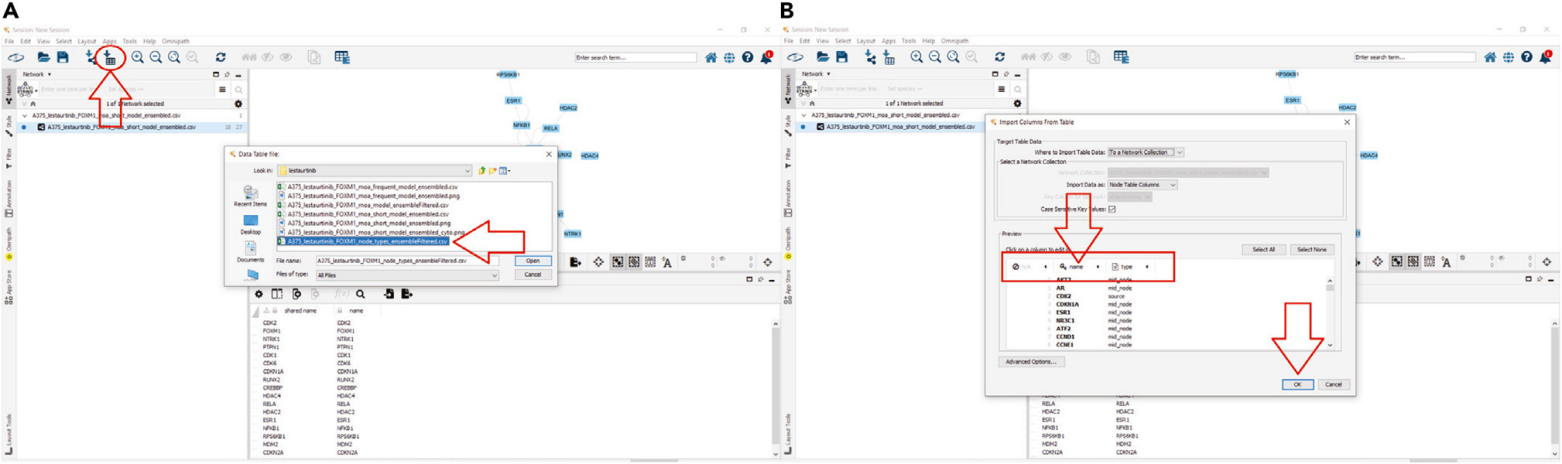
Example of importing a table with the properties of the nodes in the extracted MoA network in the Cytoscape environment, together with the step-by-step actions taken by the user to load the results These are screenshots from Cytoscape. (**A)** Loading the node table file for the ensembled reduced subnetwork. The red arrows show where the user needs to click to load the results. First, the user selects the “icon” to open a window where they can browse to find the files that contain the node properties (the second arrow). (**B)** The columns and their assigned properties when loading the CSV file. The red arrows are added to denote which column should be selected as the “key” for mapping the network and node properties, and what “icon” should they click on next.

**Figure 7:**
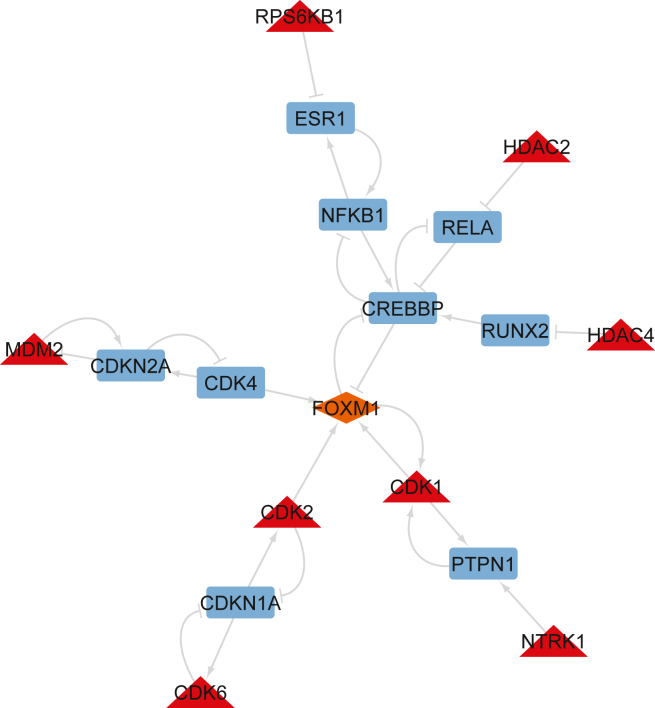
An example of a shot-paths version of a trimmed ensemble network explaining the off-target effect that leads Lestaurtinib to inhibit more FOXM1, via previously unknown drug-target interactions

***Note:*** This can be either a file with the pattern **Prefix_drug_TF_moa_model_ensembleFiltered.csv** for the full subnetwork **or Prefix_drug_TF_moa_short_model_ensembled.csv** for the shortest path network. Then load the file with the properties of the nodes (Figure 6). For interpretation, you can change the shape of edges based on their sign and color the nodes based on their type (mid_node for intermediate nodes, source for nodes that are the targets of the drugs, and target for the TF of interest). An example of a shortest path network explaining the MoA of the off-target effect of Lestaurtinib on FOXM1 is show in Figure 7.

## Expected outcomes

In this protocol, the user trains an interactome-based deep learning model to explain the MoA of a drug’s off-target effects on TFs. By following step 1,2,3 the user reconstructs a prior knowledge network of drug-target interactions and intracellular signaling (in the form of a .tsv file saved in the data folder) and infers the activity of transcription factors from gene expression data (in the form of a .tsv file saved in the data folder), which serve as the predicted output of the model in matrix format (samples(N)xTFs(T)). This is used in step 4 to train models simulating the transcriptional response of cells under drug perturbations, along with the Pearson correlation between fitted and true values of TF activity, stored in ***[CellPrefix]_trainPerformance_perTF.csv*** at a designated results’ directory. This performance metric serves as a proxy for prediction quality for each TF. The input to the joint model (drug layer and PKN-based signaling neural network) is log-transformed concertation of drugs in matrix format (samples(N)xdrugs(D). It is expected that the user generates multiple models to form an ensemble, that are stored in .pt file format. In step 5, inferred DTIs are extracted from the models, which are saved in .csv format. In step 6 well-fitted TFs with large estimated off-target effects are identified. The estimated off-target effects are stored in a .csv file and visualized (Figure 4). In step 7, sub-networks are generated describing the signal flow from the off-target effect of a specific drug to a specific TF (in our case study, Lestaurtinib’s effect on FOXM1). A full subnetwork based on the signaling activity across the ensemble of models is generated as well as a shortest path version, which is saved in .csv format. These are subsequently visualized using Cytoscape (Figure 7).^6^

## Limitations

This protocol’s main limitation (with a potential workaround) is that drugs and off-targets are limited to the drugs and targets present in the training data (e.g., for the A375 case study there are 512 drugs and 355 targets). This is a consequence of the drug module’s construction. Specifically, the models simulate drug-induced intracellular signaling and can infer the MoA of a drug’s off-target effect for a specific pre-defined drug-target space, defined and constrained by prior knowledge of drug-target interactions. However, these limitations may be overcome by future expansions of the drug module and the methodology for drug-target interaction inference is agnostic of the model architecture when using the integrated gradient-based approach. Meanwhile, a potential workaround, for the current constraint drug-target space, could be to utilize the chemical similarity of a drug of interest combined with other functional similarities (e.g., known targets, a known mechanism of action, affected downstream genes or pathways, etc.) to identify “neighbors” drugs in the training set of the model to use as proxies for getting insights for their drug of interest.

As with all deep learning methods, a limitation is that a large dataset is required to train the model. In our previous work using synthetic data, we found that at least 100 samples are needed to train predictive models.^2^ DT-LEMBAS was trained on 233 unique drug conditions, but empirically we have noticed that at least 100–120 conditions are always required; below that to train a model the user needs to select to predict much fewer TFs (currently ∼100) and with a smaller PKN. Generally, data size requirements are dependent on how many TFs are predicted and how big is also the potential targets’ space. To avoid over-fitting, you may increase the regularization of the weights, or attempt to gather more data. Lack of data results in high model uncertainty, thus making the subnetworks that explain the possible MoA of off-target effects far too comprehensive to interpret. Alleviating this may require the user to have a more thorough hyper-parameter tuning of the whole pipeline (regularization strength, number of gradient thresholds tested, error increase threshold, etc.), leading to increased computing time and resource requirements. Additionally, the trained models are cell line-specific and do not offer an immediate way for an already trained model to be used for predictions in another cell line. However, in future work, this limitation could be overcome, by using a representation of each cell type as input to condition the model.

## Troubleshooting

### Problem 1

When trying to run steps 2a and 2b you may encounter an error similar to the following.

Error: cannot allocate vector of size 3.1 Gb

Error during wrapup: cannot allocate vector of size 12.5 Gb

Error: no more error handlers available (recursive errors?); invoking ′abort′ restart

### Potential solution

This is due to either R limiting how much of the RAM in your system it can access, or insufficient RAM in general in your system. For the first, you may increase the number in the command: memory.limit(size = 16000) which will result in enabling R using more memory.

In the case the problem persists it means your computer system does not have enough RAM and you may need to switch to other options such as computer clusters.

### Problem 2

The second sub-step in step 5 may take too long to run, or regardless of the slow speed you want more granularity in calculating thresholds.

### Potential solution

You may go to line 88 of the **inferEnsembleScoreCaseStudy.py** file (or 82 for InferInteractionScoresLinAlg.py) and modify the command: thresholds = list(np.logspace(-3.5, 3.5, num = 50)).

If you decrease the `num` parameter the script will run faster, but you will lose granularity in calculating a gradient threshold. On the other hand, if granularity is the goal and the speed is not a problem you may increase the number.

### Problem 3

If in step 7, when running the **inferMoACaseStudy.py** script, you specify a frequency threshold for potential targets (--source_freq_thresh) that is too large, the algorithm may result in inferring zero potential off-targets, and thus you may encounter the following error in line 578: AssertionError: No inferred drug`s targets kept!

### Potential solution

Re-run the script after decreasing the frequency threshold when passing the argument target frequency argument: e.g., --source_freq_thresh 0.5.

## Resource availability

### Lead contact

Further information and requests for resources should be directed to and will be fulfilled by the lead contact, Avlant Nilsson (avlant.nilsson@ki.se).

### Technical contact

Questions about the technical specifics of performing the protocol should be directed to and will be answered by the technical contact, Nikolaos Meimetis (meimetis@mit.edu).

### Materials availability

This study did not generate any reagents.

### Data and code availability

• This protocol utilizes existing, publicly available data. These accession numbers for the datasets are listed in the key resources table. Specifically, the L1000 dataset^9^ was used to train the A375 model for the case study. The Broad’s Institute Repurposing Hub^11^ was used for retrieving drug-target interactions.
• All original code and steps used in this protocol have been deposited in a GitHub repository (https://github.com/Lauffenburger-Lab/DrugsANNSignaling), which is publicly available. The repository has also been archived at Zenodo (https://doi.org/10.5281/zenodo.14057135). DOIs are listed in the key resources table. In the same repository, the analyzed data that were used to train our models and produce all figures are also deposited.
• The cell-line-specific ensembles of trained DT-LEMBAS models (50 models) trained for 33 cell lines in the L1000 dataset are deposited at Zenodo (https://doi.org/10.5281/zenodo.14057298). DOIs are listed in the key resources table.

Any additional information required to reanalyze the data reported in this study is available from the lead contact upon request.

## Acknowledgments

The authors would like to thank Christine Wiggins and Luka Karginov for their extensive and valuable feedback on the software installation guide of this work. We acknowledge funding from the Swedish Cancer Society, grant no. 23 0693 JIA, and the SciLifeLab & Wallenberg Data-Driven Life Science Program, grant no. KAW 2020.0239 (A.N.). We also acknowledge funding from US ARO cooperative agreement W911NF-19-2-0026 for the Institute for Collaborative Biotechnologies (D.A.L.) and NIH contract #75N93019C00071 (D.A.L.).

## Author contributions

N.M. generated and implemented the code for this protocol, executed the simulations, preprocessed the data, and trained the final models, under the supervision of A.N. N.M. wrote the protocol and A.N. edited it. D.A.L. provided feedback for drafting the manuscript.

## Declaration of interests

The authors declare no competing interests.
